# Development of Kinematic Graphs of Median Nerve during Active Finger Motion: Implications of Smartphone Use

**DOI:** 10.1371/journal.pone.0158455

**Published:** 2016-07-01

**Authors:** Hoi-Chi Woo, Peter White, Ho-Kwan Ng, Christopher W. K. Lai

**Affiliations:** Department of Health Technology and Informatics, The Hong Kong Polytechnic University, Hung Hom, Kowloon, Hong Kong, China; University of Chicago, UNITED STATES

## Abstract

**Background:**

Certain hand activities cause deformation and displacement of the median nerve at the carpal tunnel due to the gliding motion of tendons surrounding it. As smartphone usage escalates, this raises the public’s concern whether hand activities while using smartphones can lead to median nerve problems.

**Objective:**

The aims of this study were to 1) develop kinematic graphs and 2) investigate the associated deformation and rotational information of median nerve in the carpal tunnel during hand activities.

**Methods:**

Dominant wrists of 30 young adults were examined with ultrasonography by placing a transducer transversely on their wrist crease. Ultrasound video clips were recorded when the subject performing 1) thumb opposition with the wrist in neutral position, 2) thumb opposition with the wrist in ulnar deviation and 3) pinch grip with the wrist in neutral position. Six still images that were separated by 0.2-second intervals were then captured from the ultrasound video for the determination of 1) cross-sectional area (CSA), 2) flattening ratio (FR), 3) rotational displacement (RD) and 4) translational displacement (TD) of median nerve in the carpal tunnel, and these collected information of deformation, rotational and displacement of median nerve were compared between 1) two successive time points during a single hand activity and 2) different hand motions at the same time point. Finally, kinematic graphs were constructed to demonstrate the mobility of median nerve during different hand activities.

**Results:**

Performing different hand activities during this study led to a gradual reduction in CSA of the median nerve, with thumb opposition together with the wrist in ulnar deviation causing the greatest extent of deformation of the median nerve. Thumb opposition with the wrist in ulnar deviation also led to the largest extent of TD when compared to the other two hand activities of this study. Kinematic graphs showed that the motion pathways of median nerve during different hand activities were complex.

**Conclusion:**

We observed that the median nerve in the carpal tunnel was rotated, deformed and displaced during the hand activities that people may be performed when using a smartphone, suggesting an increased risk of carpal tunnel syndrome (CTS). In addition, the kinematic graphs of median nerve developed in the present study provide new clues for further studies on the pathophysiology of CTS, and alerting smartphone users to establish proper postural habits when using handheld electronic devices.

## Introduction

Nowadays, as mobile technology continues to advance, people often use smartphones instead of computers in their daily live. It is easy to find smartphone users who are walking along the street with their eyes staring at their smartphones, and at the same time with their thumb or finger moving on the touchscreens. Indeed, smartphone brings us many conveniences. However, increasing evidence shows that prolonged use of a smartphone, together with inappropriate wrist position, may lead to repetitive strain injury of the wrist, especially when fingers, hands and wrists are overused [1].

Carpal tunnel syndrome (CTS) is a compression neuropathy that is characterized by pain, numbness and tingling over the first 3½ digits and radial portion of the palm [2,3]. The incidence rates of CTS range from 2.7% to 5.8% in the general adult population, with women being more susceptible to CTS than men by a ratio of 3:1 [4,5]. Although the pathophysiology of CTS remains unclear, the compression by surrounding tendons and soft tissue onto the median nerve at the carpal tunnel is considered as the predisposing factor for triggering CTS [2,6].

The carpal tunnel is a narrow channel in the wrist that has nine flexor tendons [one flexor pollicis longus (FPL), four flexor digitorum superficialis (FDS) and four flexor digitorum profundus (FDP) tendons] and the median nerve passes through it [7]. Geographically, it is bounded by the eight carpal bones on the dorsal side and the transverse carpal ligament (also known as the flexor retinaculum) on the volar side [3,6]. Also, there are multiple layers of subsynovial connective tissue (SSCT) in the carpal tunnel that facilitates the sliding movements of the flexor tendons and median nerve during hand activities [8,9]. Due to the limited space, median nerve in the carpal tunnel is always subjected to compression by the adjacent flexor tendons during hand activities. In general, an abnormal high compression force on median nerve will restrict nerve blood flow and oxygen tension, and subsequently lead to reversible local ischemia and impairment of nerve function [6,10]. If the compression persists, permanent damage may occur at the myelin sheaths and axons of the median nerve, leading to an alteration in the supporting connective tissue, and resulting in the development of CTS [10].

Clinical diagnosis of CTS is usually based on physical examination, electrodiagnostic tests and medical imaging. Although both nerve conduction study and electromyography are useful in localizing the site of pathology and determining the severity of the CTS condition, they are unable to provide anatomical visualization of the nerves and their surrounding structures, as well as causing discomfort due to electric shock during the procedure [11]. Therefore, diagnostic ultrasound has become an attractive complement to electrodiagnostic studies for the evaluation of peripheral nervous system. Apart from its low cost and high availability, ultrasound imaging also offers dynamic scanning and superior visualization of the median nerve and tendons in the carpal tunnel [12].

The most widely used and accepted ultrasound parameters for differential diagnosis of CTS are the cross-sectional area (CSA), swelling ratio (SR) and flattening ratio (FR) of median nerve, as well as the bowing and thickness of the transverse carpal ligament (TCL) at the carpal tunnel [13]. Recently, many researchers have made use of different medical imaging modalities to study the deformation and displacement of median nerve under various hand activities [14–23], and the majority of them opted to use ultrasound rather than other imaging techniques due to its capability for real-time dynamic scanning during hand activity [24]. Although evidence of deformation and greater extent of displacement of median nerve during hand activity are collected from these previous studies, many of them failed to monitor the dynamic change of median nerve in real-time.

Many previous studies have reported that repetitive finger motions, forceful exertion and awkward wrist posture are the risk factors of CTS [25–27]. Recent evidence has indicated that frequent smartphone users are more susceptible to have swollen median nerve, impaired hand function and reduced pinch strength [28]. In addition, a previous study suggested that median nerve would become deformed after using a smartphone for 30 minutes [29]. However, the extents of deformation, rotation and displacement of median nerve in the carpal tunnel during different hand activities are not fully understood and little is known about the kinematics of the median nerve during active finger motions. In this regard, we hypothesized hand activities that are commonly performed when using smartphones: 1) thumb opposition with wrist in neutral position, 2) thumb opposition with wrist in ulnar deviation and 3) pinch grip with wrist in neutral position, will lead to significant deformation, rotation and displacement of median nerve in the carpal tunnel. Therefore, the aims of this study were to 1) develop kinematic graphs and 2) investigate the associated deformation and rotational information of the median nerve in the carpal tunnel during hand activities that people may be performed when using a smartphone.

## Materials and Methods

### Ethics Statement

The Human Subjects Ethics Sub-committee of The Hong Kong Polytechnic University approved this study (HSEARS20131108002). Written consents were obtained from all participants after the purpose and procedures of the study were fully explained to them.

### Participants

A total of 30 apparently healthy university students (15 males and 15 females, age range 18–30 years) were successfully recruited from The Hong Kong Polytechnic University. Participants were excluded from the study if they had: 1) any history or symptoms of CTS including pain, tingling, burning, numbness, or a combination of these symptoms on the palmar aspect of the thumb, index finger, middle finger, or radial half of the ring finger [30]; 2) a history of wrist surgery including carpal tunnel injection or fracture; 3) a history of underlying disorders associated with CTS including diabetes mellitus, rheumatoid arthritis, acromegaly, hypothyroidism, pregnancy, or obesity (body mass index > 30 kg/m^2^) [31]; or 4) anatomic variations in the median nerve including bifurcation proximal to distal radio-ulnar junction, as identified during the ultrasound experiment [32]. All participants were requested to complete a questionnaire detailing their demographic data, such as age, gender, height, weight, and determination of dominant hand, as well as the presence of medical history listed in the above exclusion criteria before the ultrasound examination.

### Equipment

The ultrasound examination was carried out in the ultrasound laboratory of The Hong Kong Polytechnic University. The room temperature of the examination room was kept at 24°C. A Philips ultrasound unit (Model: HD11 XE, Philips Medical Systems, Bothell, WA, USA) with an L12–5 linear array transducer of frequency 3–12 MHz was used in this study. The image resolution, frame rate and acquisition time of the captured video were set at 320×240 pixels, 30 frames per second (fps) and 20 seconds, respectively. A trained sonographer (HW) performed all ultrasound measurements and another researcher (HN) conducted the image analysis of collected ultrasound images.

### Image Acquisition Procedure

Since our proposed hand activities required precise control of digits movement and wrist position, therefore the dominant hand of participant was selected for investigation. Participants were asked to practice our proposed hand activities for 10 to 15 minutes before the start of the ultrasound examination. All participants were requested to sit on a chair facing the examiner, with the elbow of dominant hand flexed approximately at 120°, forearm resting flat on a table, hand supinated and fingers fully extended. A pillow was placed under their arm and hand to maximize their comfort. During the ultrasound examination, participants were instructed to move their thumb and keep other parts of the hand still when performing hand activity. An ultrasound transducer was placed transversely at the level of the wrist crease (corresponding to the proximal part of the carpal tunnel), to avoid undesirable physical contact of the flexing thumb or fingers with the transducer and at the same time allowing better control of insonation angle between the ultrasound beam and the structures of interest. The lunate bone was also identified as the bony landmark for the correction of any minor hand movement that may occur during hand activity. In addition, to avoid giving compression onto the wrist and the inner structures, the ultrasound transducer was carefully held without applying any pressure on the participant’s skin, and was maintained at an angle perpendicular to the median nerve in order to minimize distortion and avoid anisotropic artifacts [12].

All participants were instructed to perform the following three hand activities in a set order: 1) thumb opposition with the wrist in neutral position, 2) thumb opposition with the wrist in ulnar deviation and 3) pinch grip with the wrist in neutral position.

**Fig 1:**
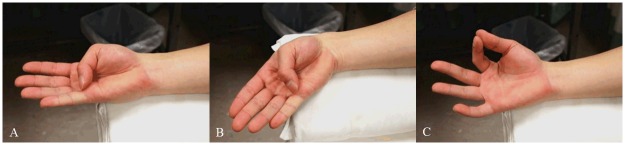
Photos of the three hand activities performed in this study. The thumb and fingers position and wrist angle at the end of motion: (A) Thumb opposition in neutral position, (B) Thumb opposition in ulnar deviation, and (C) Pinch grip.

1. Thumb opposition with the wrist in neutral position referred to flexion of 1^st^ metacarpophalangeal (MCP) joint until the thumb touched the base of little finger, which was defined as 5^th^ MCP joint and with the wrist in neutral position (Fig 1A).
2. Thumb opposition in ulnar deviation referred to flexion of 1^st^ MCP joint until the thumb touched the base of little finger while the wrist was adducted maximally (Fig 1B).
3. Pinch grip referred to flexion of 1^st^ and 2^nd^ MCP joints until the tips of the thumb and index finger were in contact and with the wrist in neutral position (Fig 1C).

Besides, they were instructed to follow a timer to complete the hand activity within 2 seconds (the first second was for the thumb to move from reposition to opposition and the latter one second was for the thumb to return back from opposition to reposition). Data from three complete cycles of thumb repetition motion were recorded in audio video interleave (AVI) video format at 30 fps for analysis.

### Image Analysis

Adobe Premiere Pro (Version 6.0.0, Adobe Systems, San Jose, California, USA) was used to capture still images in bitmap (BMP) format (the resolution and bit depth will remain unchanged) from the collected ultrasound cine-loops. In this study, only the first second of the hand activity, i.e. during thumb opposition-reposition (half cycle of the excursion) were extracted for analysis, resulting in a total of six images with equal time intervals (0.2 seconds apart from each image) being generated for each hand activity. The latter half of the video clip (when the thumb was returning back from opposition to reposition) was discarded. The six images from a single motion were labeled according to the time span of the motion, with all fingers in the natural/relaxed position defined as time point 1, and the successive frames defined as time point 2, and so forth until the end of opposition/pinch grip as time point 6.

**Fig 2:**
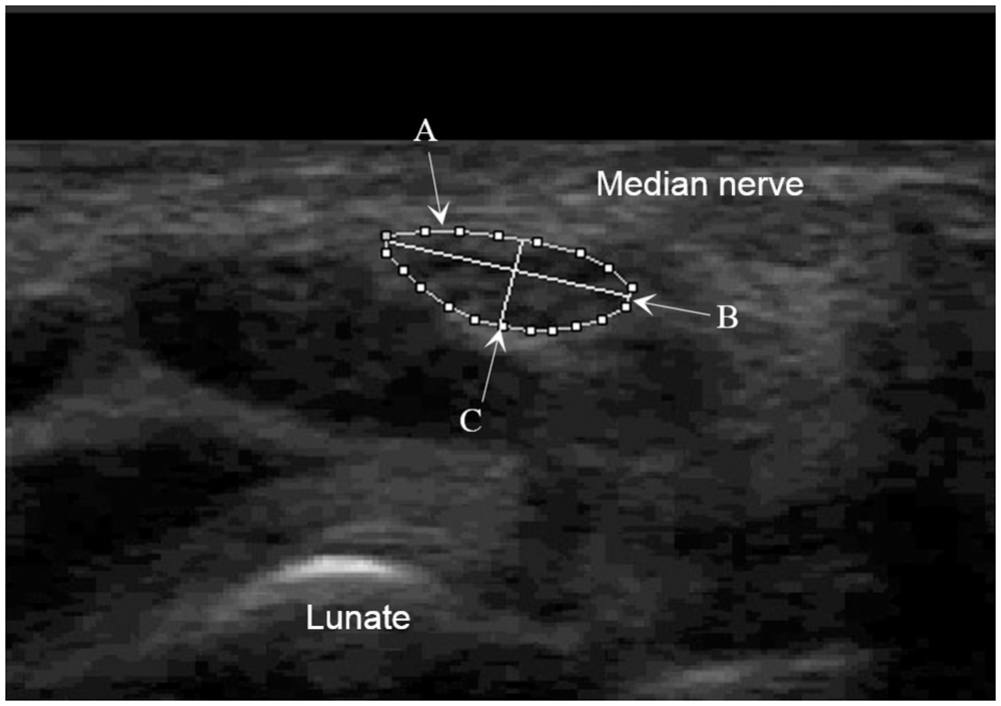
The transverse scan view of an ultrasound image of the median nerve obtained at the level of the wrist crease. The trace of the hypoechoic boundary of median nerve (A) denotes cross-sectional area of median nerve. The flattening ratio of median nerve is calculated by the ratio of B/C, where (B) denotes the major axis length and (C) denotes the minor axis length of median nerve.

Note: the coordinate of the apex of the lunate bone on each image was defined as a reference point to correct for any minor hand movements during a hand activity.

Finally, all collected images were evaluated using the image processing software ImageJ (Version 1.47, Rasband, US National Institutes of Health, Bethesda, Maryland, USA). After the pixel values were calibrated into international system of units (SI units) [33], the hypoechoic boundary and the short axis and long axis of the median nerve were outlined (Fig 2) for the determination of deformation, rotation and displacement information of median nerve during different hand activities:

1. **Cross-sectional area (CSA)** was defined as the area within the hypoechoic boundary of the median nerve by a direct tracing method.
2. **Flattening ratio (FR)** was defined as the ratio of the major axis length to the minor axis length of the median nerve.
3. **Rotational displacement (RD)** was defined as the angular displacement of the major axis of the median nerve relative to that of the immediate previous frame of a motion, where anti-clockwise direction was defined as positive.
  - **Absolute rotational displacement (ARD)** was defined as the absolute value of RD.
  - The **cumulative ARD** was defined as the sum of all averaged ARDs.
4. **Translational displacement (TD)** was defined as the distance of the centroid of median nerve travelled relative to the previous successive frame of a motion. Displacements in the radial-ulnar and palmar-dorsal directions were defined as the x-axis and y-axis, respectively in the kinetic graph, where radial and palmar directions were defined as positive.
  - **Resultant translational displacement (RTD)** was defined as the distance travelled of the centroid coordinates of the median nerve relative to immediate previous frame of a motion using pythagorean equation (although the movement of median nerve may not be linear, we assumed it to be linear for ease of calculation).
  - The **cumulative RTD** was defined as the sum of all averaged RTDs.

### Statistical Analysis

All results were analyzed using SPSS Statistics software (Version 18.0, SPSS Inc, Chicago, Illinois, USA) and expressed in mean ± standard deviation unless specified separately. A *p*-value of less than 0.05 was considered as significant. Prior to the start of the study, the power calculation test indicated that a total sample size of at least 30 subjects was required to provide 80% of power to detect a significant change in CSA at the 5% significance level. Both intra-rater and inter-rater reliability of ultrasound parameters were estimated by intra-class correlation coefficient (ICC) model 3. An ICC value of greater than 0.75 was considered as good reliability [33]. Three-way analysis of variance (ANOVA) was used to assess the effects of repetition (three cycles), time point (six time points) and task type (three hand activities) on all values of ultrasound parameters. One-way repeated measures ANOVA with post-hoc Bonferroni adjustment was used to compare the values of two successive time points of the same activities. Another one-way ANOVA test with post-hoc Bonferroni adjustment was used to compare the values of different ultrasound parameters across the three hand activities at the same time points. Finally, the eigenvector (in polar coordinates) was used to represent the sample center. The standard ellipse, representative of sample variation, and Hotelling’s confidence ellipse parameters (α = 0.05) were calculated for each motion to describe sample variation.

### Construction of Kinematic Graphs

Microsoft Office Excel 2007 (Version 12.0.6679.5000, Microsoft Corporation, Redmond, Washington, USA) was used to demonstrate the motion pathways of the median nerve during different hand activities and construct kinematic graphs.

## Results

### Reliability of Measurements

To test inter-rater reliability, two sonographers (HW and HN) independently measured the parameters of median nerve at the same level on the same day in all participants who underwent the ultrasound examination. Intra-rater reliability was also measured for each participant, and a second ultrasound test was performed an hour after the initial examination by the same sonographer (HW). The ICC values for intra-rater and inter-rater reliability of the measurement of the CSA were 0.980 and 0.942 respectively, while the FR were 0.948 and 0.862 respectively. As the movement pattern of median nerve was somewhat arbitrary, the ICC values for intra-rater and inter-rater reliability of RD and TD were range from fair to moderate (0.510 to 0.725).

### Demographic Characteristics of Participants

The demographic details of the study population are summarized in Table 1. Thirty university students with a mean age of 21.3 ± 1.9 years, and a mean body mass index of 21.21 ± 2.64 kg/m^2^ were recruited successfully. The vast majority of them were right-handed (93%).

**Table 1:** Demographic details of the participants.

	Whole group
Male to female ratio (M:F)	15:15
Age (years)	21.30±1.90
Height (m)	1.67±0.08
Weight (kg)	59.46±9.32
Body mass index (kg/m^2^)	21.21±2.64
Dominant hand (Right-handed to Left-handed ratio)	28:2

### Analysis of Three Factors (Repetition, Time Point and Task Type) on All Values of Ultrasound Parameters

All values for each participant performed each individual task with three repetitions are shown in S1–S3 Tables. There was no significant main effect of repetition on all values of ultrasound parameters: CSA [F(2,1566) = 0.015, *p* = 0.985], FR [F(2,1566) = 0.083, *p* = 0.920], RD [F(2,1566) = 1.230, *p* = 0.293], TD along X-axis [F(2,1566) = 0.066, *p* = 0.936], and TD along Y-axis [F(2,1566) = 0.228, *p* = 0.796], while there was also no significant main effect of time point on FR [F(5,1566) = 0.819, *p* = 0.536], RD [F(5,1566) = 0.133, *p* = 0.985], TD along X-axis [F(5,1566) = 0.014, *p* = 1.000], and TD along Y-axis [F(5,1566) = 0.063, *p* = 0.997]. However, there was a significant main effect of time point on CSA [F(5,1566) = 42.606, *p*<0.001], and a significant main effect of task type on all values of ultrasound parameters: CSA [F(2,1566) = 59.713, *p*<0.001], FR [F(2,1566) = 24.257, *p*<0.001], RD [F(2,1566) = 33.696, *p*<0.001], TD along X-axis [F(2,1566) = 5.127, *p* = 0.006], TD along Y-axis [F(2,1566) = 69.030, *p*<0.001]. There were no significant interaction effects of all these three factors on all values of ultrasound parameters.

### Within the Same Hand Activity: Comparison of Median Nerve Deformation Information across Different Time Points

**Fig 3:**
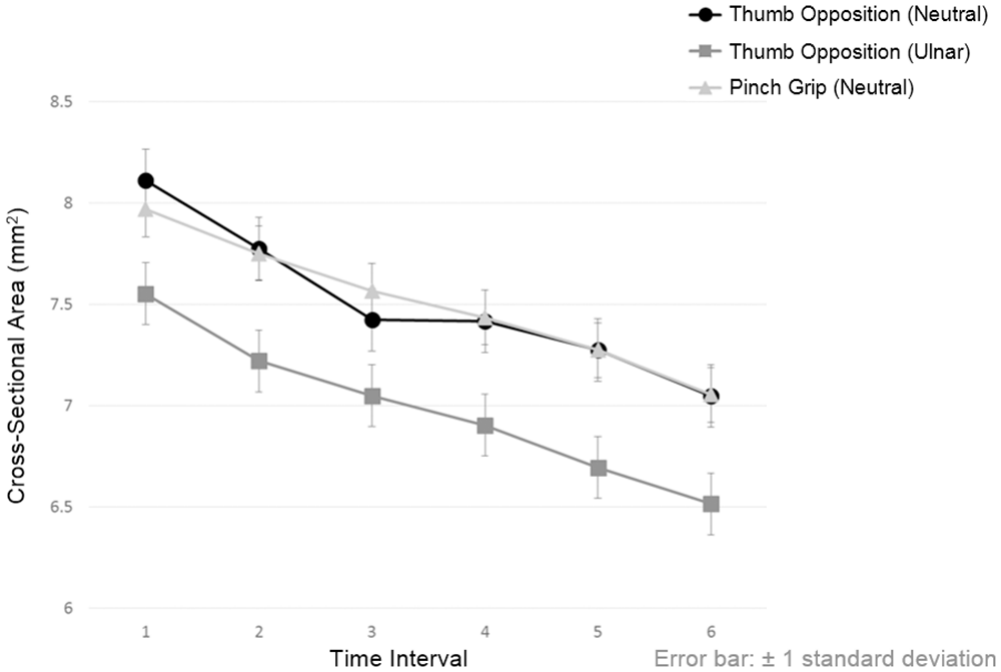
Gradual reduction in the cross-sectional area of median nerve across six successive time points when performing different hand activities. Significant differences exist between the same time points of different hand activities and between two successive time points of same hand activity after Bonferroni adjustment (*p*<0.05) are presented in S1 Fig.

**Fig 4:**
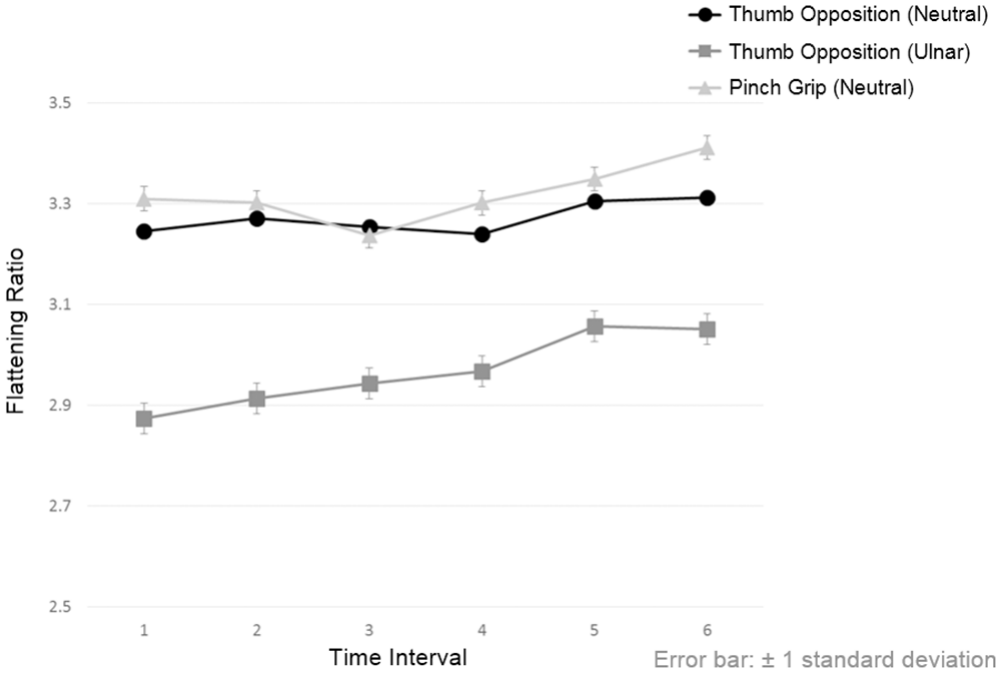
The Change of flattening ratio of median nerve across six successive time points when performing different hand activities. Significant differences exist between the same time points of different hand activities and no significant differences between two successive time points of same hand activity are presented in S2 Fig.

The CSA and FR of the median nerve across the six successive time points in different hand activities are summarized in Tables 2–4. The reduction of CSA caused by thumb opposition in neutral position, thumb opposition in ulnar deviation and pinch grip was 13%, 14% and 12%, respectively, whereas the FRs were increased by 2%, 6% and 3%, respectively. A significant gradual reduction in CSA [F(5,1566) = 42.606, *p*<0.001] (Fig 3) but not in FR [F(5,1566) = 0.819, *p* = 0.536] (Fig 4) across the successive time points during a particular hand activity was noted, except in the hand activity with thumb opposition in neutral position where there was significant reduction in CSA of the median nerve between successive time points of 1 and 2 and successive time points of 5 and 6 only.

**Table 2:** Results of median nerve deformation and displacement under thumb opposition in neutral position across six time points.

Time point	CSA (mm^2^)	FR	Relative changes when compared to the previous time point			Cumulative changes from time point 1	
			RD (°)	TD along X-axis (mm)	TD along Y-axis (mm)	ARD (°)	RTD (mm)
**1**	8.11±1.10*	3.25±0.90	----	---	---	---	---
**2**	7.77±1.01*	3.27±0.95	0.18±1.79	-0.02±0.31	-0.01±0.15	1.36±1.15*	0.35±0.25*
**3**	7.42±1.01	3.25±1.03	0.30±2.43	-0.14±0.34	-0.01±0.07	3.02±2.34*	0.69±0.42*
**4**	7.42±1.00	3.24±0.91	-0.82±2.84	-0.04±0.27	-0.01±0.09	5.13±4.09*	0.99±0.51*
**5**	7.27±1.00*	3.30±1.05	0.27±2.05	0.08±0.29	0.01±0.07	6.62±4.96*	1.31±0.63*
**6**	7.05±1.00*	3.31±1.01	-0.01±1.99	0.04±0.32	0.01±0.08	8.20±5.49*	1.63±0.80*

Where CSA = cross-sectional area; FR = flattening ratio; RD = rotational displacement; TD = translational displacement; ARD = absolute rotational displacement; and RTD = resultant translational displacement. The (*) denotes *p*<0.05 between two successive time points after Bonferroni adjustment.

**Table 3:** Results of median nerve deformation and displacement under thumb opposition in ulnar deviation across six time points.

Time point	CSA (mm^2^)	FR	Relative changes when compared to the previous time point			Cumulative changes from time point 1	
			RD (°)	TD along X-axis (mm)	TD along Y-axis (mm)	ARD (°)	RTD (mm)
**1**	7.55±0.77*	2.87±0.67	----	---	---	---	---
**2**	7.22±0.72*	2.91±0.71	-0.09±3.50	-0.02±0.22	0.03±0.10	2.46±2.45*	0.30±0.14*
**3**	7.05±0.66*	2.94±0.71	-0.27±2.84	-0.03±0.31	-0.01±0.09	4.50±3.37*	0.68±0.35*
**4**	6.90±0.69*	2.97±0.67	0.12±2.53	-0.01±0.42	-0.02±0.10	6.24±4.44*	1.09±0.54*
**5**	6.70±0.69*	3.06±0.68	0.07±2.78	-0.09±0.40	-0.04±0.10	8.22±4.91*	1.54±0.74*
**6**	6.52±0.70*	3.05±0.64	-0.34±2.13	-0.06±0.33	0.04±0.14	9.79±5.50*	1.89±0.86*

Where CSA = cross-sectional area; FR = flattening ratio; RD = rotational displacement; TD = translational displacement; ARD = absolute rotational displacement; and RTD = resultant translational displacement. The (*) denotes *p*<0.05 between two successive time points after Bonferroni adjustment.

**Table 4:** Results of median nerve deformation and displacement under pinch grip across six time points.

Time point	CSA (mm^2^)	FR	Relative changes when compared to the previous time point			Cumulative changes from time point 1	
			RD (°)	TD along X-axis (mm)	TD along Y-axis (mm)	ARD (°)	RTD (mm)
**1**	7.97±0.96*	3.31±0.88	----	---	---	---	---
**2**	7.75±0.93*	3.30±0.90	-0.34±2.11	0.05±0.20	0.03±0.16	1.59±1.39*	0.28±0.18*
**3**	7.57±0.90*	3.24±0.84	-0.52±2.26	0.02±0.14	-0.04±0.16	3.27±2.53*	0.52±0.32*
**4**	7.44±0.90*	3.30±0.92	-0.07±1.25	-0.01±0.23	0.01±0.08	4.20±2.52*	0.81±0.32*
**5**	7.27±0.85*	3.35±0.89	0.36±1.78	-0.03±0.27	0.01±0.08	5.71±3.02*	1.12±0.36*
**6**	7.05±0.87*	3.41±0.96	0.79±2.07	-0.01±0.28	-0.01±0.06	7.42±3.63*	1.39±0.43*

Where CSA = cross-sectional area; FR = flattening ratio; RD = rotational displacement; TD = translational displacement; ARD = absolute rotational displacement; and RTD = resultant translational displacement. The (*) denotes *p*<0.05 between two successive time points after Bonferroni adjustment.

### Across Different Activities: Comparison of Median Nerve Deformation Information at the Same Time Points

At the same time points across different hand activities, significant differences were found in CSA [F(2,1566) = 59.713, *p*<0.001] and FR [F(2,1566) = 24.257, *p*<0.001] of the median nerve. Comparatively, thumb opposition in ulnar deviation caused the greatest reduction of CSA when compared to the other two hand activities at any same time points (Fig 3).

### Median Nerve Rotation, Displacement and Kinematic Graphs

The RD, TD, ARD and RTD information of the median nerve when performing the three hand activities across six successive time points are summarized in Table 2. During each hand activity, the median nerve demonstrated only a small degree of rotation by less than 1°. Overall, thumb opposition in ulnar deviation resulted in the longest distance traveled of median nerve when compared to the other two activities, with pinch grip led to the shortest displacement of median nerve.

**Fig 5:**
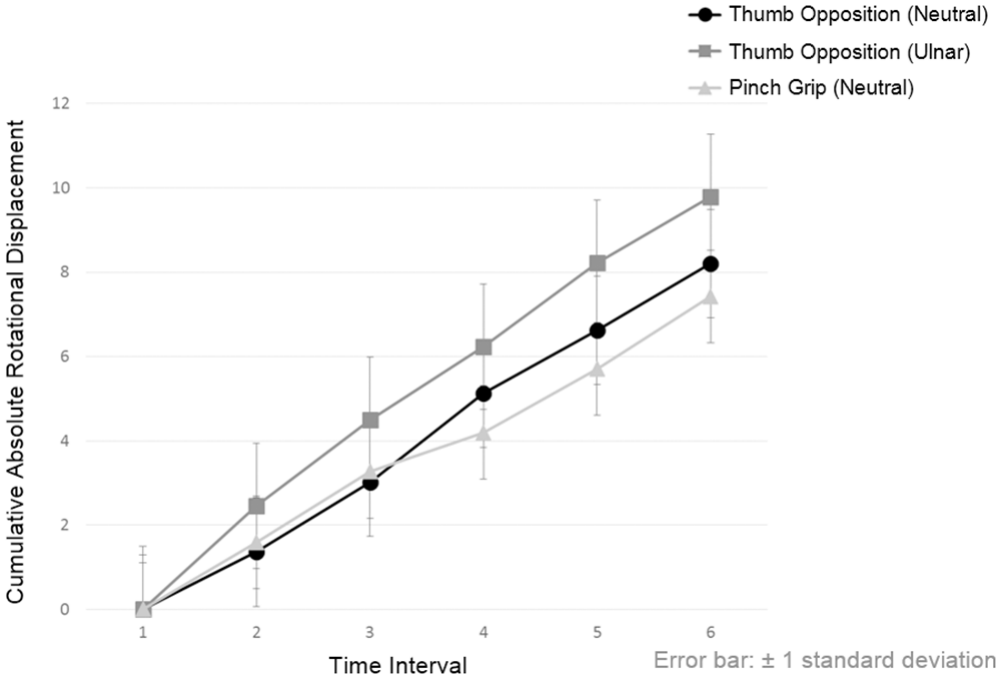
The cumulative absolute rotational displacement of median nerve across six successive time points when performing different hand activities. Significant differences exist between the same time points of different hand activities and between two successive time points of same hand activity after Bonferroni adjustment (*p*<0.05) are presented in S3 Fig.

**Fig 6:**
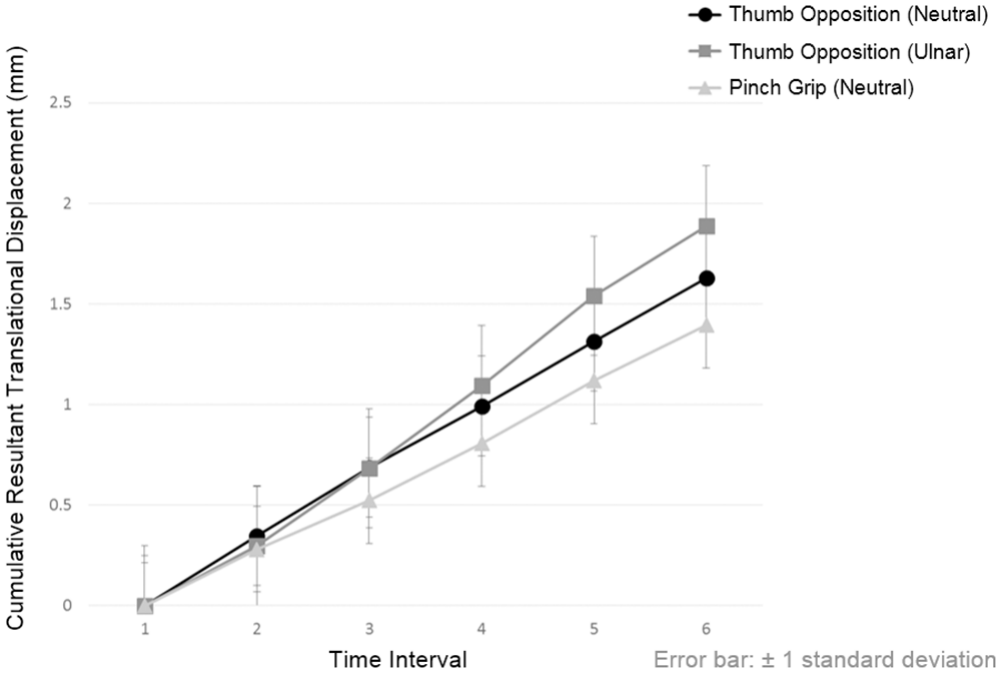
The cumulative resultant translational displacement of median nerve across six successive time points when performing different hand activities. Significant differences exist between the same time points of different hand activities and between two successive time points of same hand activity after Bonferroni adjustment (*p*<0.05) are presented in S4 Fig.

The total RTD of median nerve when performing thumb opposition in neutral position, thumb opposition in ulnar deviation and pinch grip were 1.63 mm, 1.89 mm and 1.39 mm, respectively. In these three hand activities, although the values of RD and TD along X-axis and Y-axis of median nerve at each time points were similar [F(5,1566) = 0.133, *p* = 0.985; F(5,1566) = 0.014, *p* = 1.000; F(5,1566) = 0.063, *p* = 0.997, respectively], their cumulative ARD (Fig 5) and cumulative RTD (Fig 6) across successive time points were significantly different [F(5,1566) = 227.391, *p*<0.001; F(5,1566) = 252.504, *p*<0.001, respectively].

**Fig 7:**
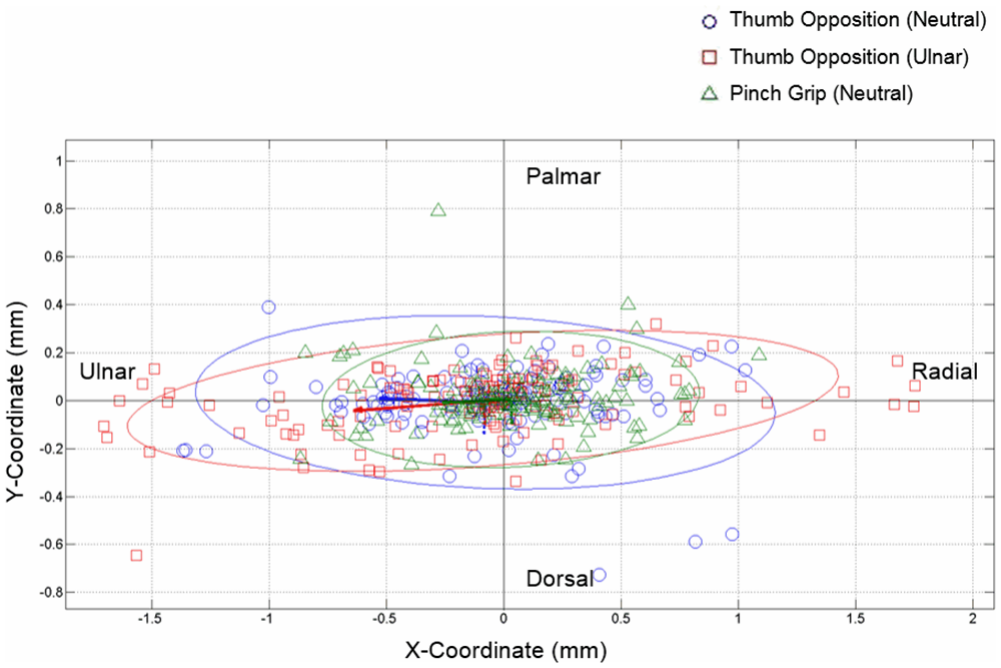
Median nerve displacement vectors across the six successive time points under different hand activities. Blue points represent thumb opposition in neutral position, red points represent thumb opposition in ulnar deviation, and green points represent pinch grip. Solid ellipses represent 95% confidence limits and the lines represent the eigenvectors for each motion.

**Fig 8:**
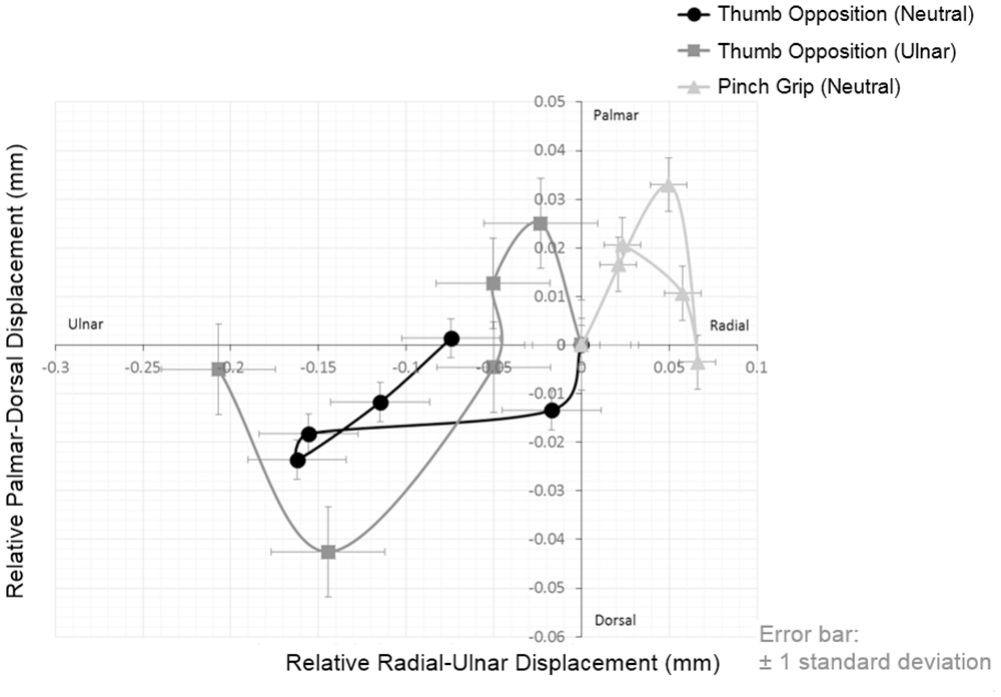
Median nerve kinematic changes across the six successive time points under different hand activities. Mean values of the motion pathways of the median nerve of all subjects are shown.

The individual and mean values of the motion pathways of the median nerve for all subjects are plotted in Figs 7 and 8 (S5 and S6 Figs), respectively. The plot indicated that the motion pathways of the median nerve were not straight lines but rather complicated curves. Among individuals, the median nerve displacement vectors showed a wide range of variation in both directions and magnitude of movement even when individually performing the same hand activity.

## Discussion

In this study, the deformation, rotation and displacement information of median nerve under the conditions of thumb opposition in neutral position, thumb opposition in ulnar deviation and pinch grip were reviewed quantitatively in a dynamic manner. In addition, kinematic graphs were developed and showed the complex motion of median nerve when performing different hand activities.

### Dynamic Changes of Median Nerve in the Carpal Tunnel during Hand Activities

The significant reduction in the CSA of median nerve during hand activities may probably due to the movement of the adjacent flexor tendons [17,19,22]. During hand activities, the FPL, FDS and FDP muscles need to contract and pull the tendons to flex the thumb and fingers [6]. As the tendons and median nerve is freely to move in a three dimensional plane, the tendons not only can passively move longitudinally, but also can slide transversely within the narrow space in the carpal tunnel [19]. Due to the narrow space available within the carpal tunnel, these tendons usually move towards the median nerve and, thereby, compressing onto the median nerve. Since the median nerve is a mobile structure, it will stretch and glide passively in response to the sudden changes in the positions of these adjacent structures [22]. Such a direct contact between the median nerve and the surrounding tendons is expected to be the primary source of median nerve compression [34], and the persistent and repetitive compression will increase the risk of developing CTS [10,28,29].

### Median Nerve Deformation

A previous study found that the CSA of median nerve was reduced after using a smartphone for 30 minutes, and suggested that the reduced space in the carpal tunnel was due to thickened muscles and ligaments after the use of smartphones. [29]. Another similar study also found that the FPL tendons were thickened at the mid-thenar and MCP joint levels after using smartphone [28]. When performing pinch grip and thumb opposition with the wrist in ulnar deviation, a gradual reduction in the CSA of median nerve was noted in the present study. It may be due to the enlargement of tendons and soft tissues in the carpal tunnel that reducing the amount of space available for the movement of median nerve. Most previous studies analyzed the initial and final frames of motion that underestimate the complexity of the movement of median nerve and lead to inaccurate results [17–23]. When taking the initial and final frames of motion for comparison, our result was consistent with these previous studies that suggesting CSA of the median nerve was smaller in thumb opposition than in thumb reposition, and had no significant differences in FR during a thumb opposition [17,19]. Interestingly, significant reduction in CSA was found only at the start and end positions of the motion during thumb opposition in neutral position, this observation may imply that a larger force may be applied at the start and end of thumb opposition causing a significant median nerve compression at these time points.

Among the three hand activities, thumb opposition with the wrist in ulnar deviation causing the greatest reduction in CSA at all time points, indicating that the greatest compression force exerting onto the median nerve. This was consistent with previous findings that finger motion and the wrist posture could both lead to median nerve deformation [20,21]. Some researchers have qualitatively described the tendons was forced to displace ulnarly while the median nerve was forced to displace radially under wrist ulnar deviation [20]. As these tendons move towards the median nerve under wrist ulnar deviation, a greater extent of reduction in CSA is expected when compared to neutral wrist position [34]. Another study also demonstrated that the contact pressure on the median nerve was increased markedly with the wrist ulnar deviation angle [35].

### Median Nerve Displacement and Rotation

Previous studies have shown that the problem of median nerve deformation and displacement were more severe in CTS patients when compared to healthy subjects [17,23,36,37]. Apart from evidence of median nerve deformation, motion pathways were also studied through kinematic graphs. In the three hand activities, the motion pathways of the median nerve were found complex. We observed that the RD and TD of the median nerve between successive time points varied in both directions and magnitude in this study, and the displacement of median nerve was considered as the consequence of the movement of the adjacent flexor tendons [20]. In the majority of cases, the median nerve moved towards the radial-palmar side with either clockwise or anti-clockwise rotation during for pinch grip, and moved towards the ulnar-dorsal side with either anti-clockwise or clockwise rotation during thumb opposition in neutral position. Conversely, the median nerve mainly moved to the ulnar side with slight clockwise rotation in either palmar or dorsal direction for thumb opposition in ulnar deviation. Our findings were consistent with a previous study that the median nerve moved towards the ulnar and palmar directions in response to thumb motion [18]. However, their study design is limited by unable to track the whole nerve pathway during motion.

The median nerve and tendons are surrounded by a filmy and supple layer of the SSCT in the healthy carpal tunnel, which facilitates smooth gliding of the median nerve [20,21]. During motion, the median nerve moved to escape the severe compression rather than remaining in situ [17]. In our study, a longer travel distance of median nerve was found in thumb opposition with ulnar deviation when compared to thumb opposition with neutral position. A longer travelled distance by the median nerve may result in greater friction between the median nerve and tendons. This may be due to the fact that the median nerve was try to escape from a location with severe compression that exerted by the adjacent flexor tendons in the narrow carpal tunnel [17]. This mobility of the median nerve may suggest a mechanism to avoid direct compression by the tendons during finger and wrist movements [36]. However, the present study did not measure the motion patterns of the flexor tendons and thus the dynamic changes in the carpal tunnel remain unclear and further research is required.

### Limitations of the Study

This study has several limitations. First, only young subjects were recruited. Bathala et al. reported that there were significant differences in the CSA of median nerve between Asian subjects aged 16–40 and over 41 years [38]. Since such differences might cause different extent of median nerve deformation and displacement, our findings may only represent the young adult population, but not the entire adult population. Second, no CTS subjects were recruited in the present study. Third, only the motion patterns of the median nerve were investigated, but movements of the flexor tendons were not measured. Although the captured images also included the tendons, the motion of the tendons could not be analyzed as the transducer was held perpendicular to the median nerve, but not to the tendons. In order to avoid anisotropy, it is suggested that the median nerve and tendons should be scanned separately, and their motion pathways should be integrated into a single model in order to yield a comprehensive picture of dynamic changes in the carpal tunnel. Finally, as we aimed to investigate the compression of the median nerve by the adjacent flexor tendons during thumb opposition, only half cycle of thumb motion was analyzed, and the remaining half cycle from opposition to reposition was discarded. In order to have a better understanding of the median nerve kinematic changes, it is suggested that an entire cycle of thumb opposition-reposition could be analyzed in the future. Based on our simulated hand movements of smartphone use, this study therefore can be used for reference and further study can compare the present results from an actual use of a smartphone.

### Clinical Implications

The strength of this study is the use of dynamic ultrasound imaging to evaluate the deformation, rotation and displacement of median nerve during hand activities quantitatively in a dynamic manner. Studying median nerve deformation, rotation and displacement are considered important in understanding the kinematics of the median nerve and assessing risks of CTS for a particular hand activity [21,23].

In addition to understanding the median nerve kinematic changes, the present study also provides ergonomic hazard to alert smartphone users to avoid using their mobile devices with their wrist in ulnar direction. In addition, smartphone manufacturers can help by avoiding the overuse of wrist ulnar deviation and optimize the range of thumb movements when using their products.

## Conclusion

The hand activities that are commonly performed when using smartphones (thumb opposition in neutral position, thumb opposition in ulnar deviation and pinch grip) cause median nerve compression and displacement, and therefore potentially contribute to increased risk of CTS. Among the three hand activities studied in this study, thumb opposition in ulnar deviation causes the most severe deformation of median nerve and hence it is strongly advised not to hold the mobile device in this manner. Kinematic graphs were successfully developed in this study and demonstrated a complex motion pathway during hand activities. Further study is warranted to develop new method for the diagnosis of CTS using dynamic ultrasound.

## Supporting Information

S1 Fig The cross-sectional area of median nerve across six successive time points when performing different hand activities. (XLSX)

S2 Fig The flattening ratio of median nerve across six successive time points when performing different hand activities. (XLSX)

S3 Fig The cumulative absolute rotational displacement of median nerve across six successive time points when performing different hand activities. (XLSX)

S4 Fig The cumulative resultant translational displacement of median nerve across six successive time points when performing different hand activities. (XLSX)

S5 Fig Median nerve displacement vectors across the six successive time points under different hand activities. (XLSX)

S6 Fig Median nerve kinematic changes across the six successive time points under different hand activities. (XLSX)

S1 Table Raw data for each participant performed thumb opposition with wrist in neutral position. (XLSX)

S2 Table Raw data for each participant performed thumb opposition with wrist in ulnar deviation. (XLSX)

S3 Table Raw data for each participant performed pinch grip with wrist in neutral position. (XLSX)
